# Association between Inflammatory and Metabolic Biomarkers and Common Mental Disorders among Adults: 2015 Health Survey of São Paulo, SP, Brazil

**DOI:** 10.3390/metabo14100535

**Published:** 2024-10-05

**Authors:** Letícia do Nascimento Maximiano Ferreira, Regina Mara Fisberg, Flavia Mori Sarti, Marcelo Macedo Rogero

**Affiliations:** 1 Department of Nutrition, School of Public Health, University of São Paulo, 715 Dr. Arnaldo Avenue, São Paulo 01246-904, SP, Brazil; leticia.nascimento.ferreira@usp.br (L.d.N.M.F.); rfisberg@usp.br (R.M.F.); 2 School of Arts, Sciences and Humanities, University of São Paulo, 1000 Arlindo Bettio Avenue, São Paulo 03828-000, SP, Brazil; flamori@usp.br

**Keywords:** common mental disorders, inflammatory biomarkers, C-reactive protein, cardiometabolic biomarkers, metabolic syndrome

## Abstract

Recent studies suggest that plasma inflammatory biomarker concentrations may represent valuable indicators for the diagnosis and prognosis of mental disorders. At the same time, metabolic alterations may contribute to the development and progression of systemic low-grade inflammation. **Background/Objectives**: This study evaluated the association between plasma inflammatory biomarkers and common mental disorders (CMD), exploring the relationship between metabolic biomarkers, metabolic syndrome (MetS), and inflammatory biomarkers in younger and older adults. **Methods**: This cross-sectional study used data from the 2015 Health Survey of São Paulo with a Focus on Nutrition Study. The occurrence of CMD was assessed through the Self-Reporting Questionnaire (SRQ-20). Blood samples were used to measure plasma concentrations of inflammatory and cardiometabolic biomarkers. MetS was defined according to the International Diabetes Federation Consensus. The Mann–Whitney test compared inflammatory biomarker concentrations across CMD groups and cardiometabolic conditions, and logistic regression models explored associations between inflammatory biomarker concentration and CMD. **Results**: The sample included 575 participants, 22.6% (n = 130) of whom had CMD. Concentrations of plasminogen activator inhibitor 1, C-reactive protein (CRP), and the systemic low-grade inflammation score varied significantly among CMD groups. CRP concentrations were positively associated with the presence of CMD, independent of confounding factors. Participants with insulin resistance, dyslipidemia, and MetS exhibited significantly higher CRP concentrations than individuals without these conditions. **Conclusions**: The findings suggest that increased plasma CRP concentrations may be a potential risk factor for CMD. Higher CRP concentrations were observed in individuals with insulin resistance, dyslipidemia, and MetS. Future interventional studies should explore these hypotheses in diverse populations.

## 1. Introduction

An increasing number of studies have been showing the effects of plasma inflammatory marker concentrations, such as C-reactive protein (CRP), interleukin (IL)-6, and tumor necrosis factor-alpha (TNF-α), in the occurrence of mental disorders, suggesting their utility for diagnosis, prognosis, and disease progression monitoring [1]. Systemic low-grade inflammation has been more frequently observed in subgroups of patients with more severe psychopathological symptoms, greater treatment resistance, and worse clinical course of mental disorders [2]. Psychosocial and biological stressors stimulate inflammatory signaling pathways, including the NOD-like receptor 3 (NLRP3) inflammasome, and the nuclear factor kappa B (NF-κB) pathway, resulting in the release of proinflammatory cytokines, such as IL-1β, IL-6, and TNF-α [3]. These cytokines can cross the blood–brain barrier and stimulate microglial cells, altering neurotransmitter synthesis [4]. Chronic stress also triggers glucocorticoid resistance, further exacerbating inflammation and contributing to the development and persistence of depressive and anxiety disorders [5].

Chronic hyperglycemia, dyslipidemia, and other metabolic alterations may lead to the development and progression of systemic low-grade inflammation [6]. The reverse is also true regarding the role of inflammation in the pathophysiology of hyperglycemia, creating a bidirectional cycle [7]. In individuals with chronic dyslipidemia and type 2 diabetes, lipoxidation and glycoxidation end-products and reactive oxygen species (ROS) stimulate immune cells and resident vascular cells to release cytokines by activating inflammatory pathways [8]. The binding of inflammatory cytokines, such as TNF-α and IL-1, along with free fatty acids, to their receptors, tumor necrosis factor receptor (TNFR), interleukin-1 receptor (IL-1R), and Toll-like receptor (TLR) on the cell membrane triggers the activation of kinases like inhibitory kappa B kinase (IKK) and c-Jun N-terminal kinase (JNK), as well as inflammasomes. These processes interfere with insulin signaling by altering the function of insulin receptors and subsequent signaling proteins [7]. Metabolic syndrome (MetS), characterized by a cluster of metabolic dysregulations, such as abdominal obesity, hypertension, hyperglycemia, and dyslipidemia, leads to systemic low-grade inflammation primarily through the increased production of proinflammatory cytokines, such as IL-6, CRP, and TNF-α [9]. These cytokines, released in response to hyperglycemia and increased adiposity, promote the activation of inflammatory pathways driving the inflammatory response associated with MetS [10].

Given the need for further research to confirm preliminary findings on the subject, and to foster potential advancements that this approach could offer for the treatment of individuals with mental disorders, this study aimed to (1) compare plasma concentrations of inflammatory biomarkers in individuals with and without common mental disorders (CMD); (2) investigate the association of differentially expressed inflammatory markers and the presence of CMD, independent of confounding factors; and (3) examine the relationship between metabolic biomarkers, MetS, and inflammatory markers previously linked to CMD. We hypothesized that plasma inflammatory biomarkers would be elevated in participants with CMD compared to individuals without this condition, and differentially expressed inflammatory markers would be associated with CMD due to the neuroinflammatory processes involved in mental disorders [3]. Additionally, we expected that cardiometabolic biomarkers and MetS would be associated with plasma inflammatory biomarkers [10].

## 2. Materials and Methods

### 2.1. Study Design and Participants

This study used data from the 2015 Health Survey of São Paulo with a Focus on Nutrition Study (2015 ISA-Nutrition). This is a population-based cross-sectional study of residents of permanent private households in the urban area of São Paulo city, collected from February 2015 to February 2016. Further details about the survey design and sample of the 2015 ISA-Nutrition were previously published elsewhere [11]. The present study included data from adults aged 20 years or above with complete information for plasma inflammatory biomarkers and CMD. The subsample was selected based on the following exclusion criteria: the presence of acute inflammatory diseases or cancer and the use of medications that could interfere with the markers of interest, including antibiotics, anti-inflammatories, immunomodulatory drugs, and antiretrovirals. Furthermore, the 2015 ISA-Nutrition study excluded individuals with chronic alcoholism, individuals on enteral or parenteral nutrition, and pregnant or lactating women. The 2015 ISA-Nutrition was approved by the Research Ethics Committee of the School of Public Health of the University of São Paulo, protocol 30848914.7.0000.542. Written informed consent was obtained from all participants.

### 2.2. Data Collection

Sociodemographic characteristics, alcohol consumption, and smoking were self-reported during the initial interview of the 2015 ISA-Nutrition survey. Anthropometric measurements followed the World Health Organization (WHO) guidelines [12]. Average weight and height values were used to calculate body mass index (BMI), defined as body mass in kilograms divided by the square of height in meters (kg/m²). BMI classification was performed according to age-specific criteria based on WHO and Pan American Health Organization standards [12,13]. Physical activity levels were evaluated using the International Physical Activity Questionnaire (IPAQ) [14].

#### 2.2.1. Common Mental Disorders

CMD involve a spectrum of symptoms, including depressive–anxious mood, low energy levels, somatic complaints, and depressive thoughts, which do not meet the full diagnostic criteria for psychiatric disorders as defined by the Diagnostic and Statistical Manual of Mental Disorders (DSM) [15,16]. The application of psychiatric screening instruments is a cost-effective and easily implemented strategy to facilitate the early detection of these symptoms [17]. Considering this scenario, the WHO endorses the Self-Reporting Questionnaire (SRQ-20) as a reliable screening tool for detecting CMD in the general population [17].

The presence of CMD was determined using the SRQ-20 validated for the Brazilian population [18]. This instrument consists of 20 closed-ended questions designed to identify symptoms of depression and anxiety experienced in the 30 days before the interview [17]. For female participants, a cutoff score of 8 or higher was used to indicate the presence of CMD, as this threshold demonstrated higher sensitivity (86%) and specificity (77%). Conversely, for male participants, a cutoff score of 6 or higher was employed, yielding better sensitivity (89%) and specificity (81%) [18]. Furthermore, for participants aged 65 years and older, a cutoff score of 5 or higher was applied, based on findings from a study of the Brazilian population that reported optimal sensitivity (76.1%) and specificity (74.6%) for both sexes at this threshold [19].

#### 2.2.2. Blood Samples

Fasting blood samples were collected from participants through venipuncture using sterile, disposable syringes by trained professionals at the participants’ homes, following prearranged appointments. Participants were instructed to abstain from alcohol for 72 h before the blood draw and to avoid intense physical activity on the day before and the day of the blood collection. The blood samples were transported to the Laboratory of Nutritional Genomics and Inflammation at the School of Public Health, University of São Paulo, in Styrofoam containers with recyclable ice packs. For each participant, nine aliquots were immediately analyzed in the laboratory, and approximately 25 additional aliquots were stored under conditions appropriate for each assay at either −20 °C or −70 °C. Specific standardized procedures were then performed, as described by Fisberg et al. (2018) [11].

#### 2.2.3. Inflammatory Biomarkers

Plasma concentrations of IL-1β, IL-6, IL-10, TNF-α, monocyte chemotactic protein1 (MCP-1), plasminogen activator inhibitor1 (PAI-1), soluble intercellular adhesion molecule-1 (sICAM-1), soluble vascular cell adhesion molecule-1 (sVCAM-1), adiponectin, and leptin were measured using a multiplex immunoassay kit (Milliplex, Merck Millipore, Burlington, MA, USA). CRP plasma concentrations were quantified using an ELISA kit (Enzyme-Linked Immunosorbent Assay, Cat. No. HEA821Hu; Cloud-Clone Corp., Houston, TX, USA).

#### 2.2.4. Systemic Low-Grade Inflammation Score

The systemic low-grade inflammation score (SIS) was computed using plasma concentrations of inflammatory biomarkers, following the methodology by Tabung et al. (2016) [20]. This score was calculated by summing the log-transformed values of the biomarkers’ standardized Z-scores. Biomarkers with anti-inflammatory properties (adiponectin and IL-10) were given negative weights. The SIS serves as an indicator of systemic low-grade inflammation in individuals. Higher (more positive) scores suggest a more proinflammatory state, whereas lower (more negative) scores indicate a more anti-inflammatory state.

#### 2.2.5. Cardiometabolic Biomarkers

The cardiometabolic biomarkers were determined by blood samples as follows: plasma glucose concentrations in sodium fluoride were determined using the colorimetric enzymatic glucose oxidase assay (Trinder reaction) (Cobas; Roche Diagnostics GmbH, Mannheim, BW, Germany); plasma insulin in ethylenediaminetetraacetic acid (EDTA) was measured using a multiplex immunoassay (LINCOplex^®^; Linco Research Inc., St. Charles, MO, USA); total serum cholesterol was analyzed using the Trinder reaction (cholesterol oxidase) (Cobas; Roche Diagnostics GmbH, Mannheim, BW, Germany); low-density lipoprotein cholesterol (LDL-c) and high-density lipoprotein cholesterol (HDL-c) were assessed using a homogeneous enzymatic colorimetric assay (Cobas; Roche Diagnostics GmbH, Mannheim, BW, Germany); serum triglycerides (TG) was measured with an enzymatic colorimetric assay (glycerol phosphate peroxidase) (Cobas; Roche Diagnostics GmbH, Mannheim, BW, Germany); and non-high-density lipoprotein cholesterol (non-HDL-c) was determined as the difference between values of total cholesterol and HDL-c.

Type 2 diabetes was determined by a fasting glycemia greater than 126 mg/dL or the use of hypoglycemic agents or insulin therapy [21]. Systemic arterial hypertension was assessed based on systolic blood pressure equal to or greater than 140 mmHg or diastolic blood pressure equal to or greater than 90 mmHg, or the use of antihypertensive medication, including hypotensive agents and diuretics [22]. Dyslipidemia was evaluated by the use of lipid-lowering medication or the presence of any of the following conditions: (1) isolated hypercholesterolemia, defined as LDL-c equal to or greater than 160 mg/dL; (2) isolated hypertriglyceridemia, defined as TG equal to or greater than 150 mg/dL; (3) mixed hyperlipidemia, defined as the presence of both of the previous criteria; and (4) HDL-c lower than 40 mg/dL in men and lower than 50 mg/dL in women, either in isolation or in combination with elevated LDL-c or TG [23].

Insulin resistance was assessed using the Homeostasis Model Assessment for Insulin Resistance (HOMA-IR). This index was derived by multiplying fasting blood glucose (mg/dL) by fasting plasma insulin (μIU/mL) and dividing the product by 405. The threshold for HOMA-IR was set at 2.71, based on the guidelines established by Geloneze et al. (2009), which are validated for the Brazilian population [24].

#### 2.2.6. Metabolic Syndrome

MetS was defined as the presence of 3 out of the following 5 criteria: (1) elevated glucose concentration (fasting plasma glucose ≥100 mg/dL) or treatment with anti-hyperglycemic medications; (2) elevated serum TG concentration (≥150 mg/dL) or treatment with anti-hypertriglyceridemia medications; (3) reduced serum HDL-c concentration (<50 mg/dL in women and <40 mg/dL in men); (4) elevated blood pressure (≥130/85 mmHg) or treatment with antihypertensive medications; and (5) central obesity (waist circumference ≥80 cm in women or ≥90 cm in men) [9].

### 2.3. Statistical Analysis

Continuous variables are presented as median, minimum, and maximum values, considering the absence of parametric distribution (according to Shapiro–Wilk test), and categorical variables are presented as absolute frequency and percentage. The Mann–Whitney test was used to evaluate differences in the concentration of inflammatory biomarkers, cardiometabolic biomarkers, and MetS between participants with and without CMD.

The analysis of associations between the inflammatory biomarkers and the presence of CMD was performed using a multivariable logistic regression model. The model was adjusted according to the following variables: demographic characteristics (age and sex assigned at birth), lifestyle factors (weekly physical activity, alcohol consumption, and smoking), and health conditions (BMI, type 2 diabetes, systemic arterial hypertension, and dyslipidemia). The analyses were restricted to subjects with complete information for the outcome and exposure variables in the study. All statistical analyses were performed using Stata, version 14, with a significance level set at 0.05.

## 3. Results

The study sample consisted of 575 participants, comprising 50.4% male with a median age of 59 years. Regarding health habits, most participants neither consumed alcohol (70%) nor smoked (83%), and the median practice of physical activity per week was 480 min. Additionally, 52% of the participants were overweight or obese, 21.2% had a previous diagnosis of type 2 diabetes, 54.6% had systemic arterial hypertension, and 71% exhibited dyslipidemia. Finally, 22.6% of the participants had CMD. Table 1 provides a detailed overview of the demographic and health characteristics of the sample.

We observed differences in the median values of inflammatory biomarkers according to the presence of CMD (Table 2). Significantly higher plasma concentrations of PAI-1 and CRP and elevated SIS were found in participants with CMD compared to individuals without this condition. No significant differences between groups were found for the other inflammatory biomarkers. Furthermore, CRP was positively associated with CMD in the multivariable logistic model, indicating that there was a 54% increase in the odds of CMD for each increase of one milligram per liter (mg/L) in plasma CRP concentrations, independent of sex, age, physical activity, alcohol consumption, smoking, BMI, type 2 diabetes, systemic arterial hypertension, and dyslipidemia (Table 3).

Significant differences in CRP concentrations were observed across cardiometabolic status and MetS. Specifically, groups of individuals with higher HOMA-IR values and serum TG concentrations exhibited increased plasma CRP concentrations. These findings indicated an association between insulin resistance, dyslipidemia, and higher CRP concentrations. Additionally, participants with MetS had significantly higher levels of CRP compared to individuals without this condition (Table 4).

## 4. Discussion

The findings of this study indicated that individuals with CMD presented increased plasma concentrations of PAI-1, CRP, and SIS compared to individuals without CMD. The elevated concentration of CRP was associated with higher odds of developing CMD. Additionally, individuals with insulin resistance, dyslipidemia, and MetS showed higher plasma CRP concentrations than participants without these diagnoses.

A meta-analysis of inflammatory markers in clinically significant symptoms of depression and anxiety found higher standardized mean differences in the levels of IL-6, IL-17A, IL-12, IL-13, neutrophils, and monocytes, as well as lower levels of lymphocytes in individuals with clinically significant depression. In those with clinically significant anxiety, higher levels of basophils, CD45RA+, and CD62L+ were observed, alongside lower levels of eosinophils and IL-12 [1]. There are several hypotheses suggesting the linking of systemic low-grade inflammation with mental disorders. The immune activation by psychosocial and biological stressors leads to the release of damage-associated molecular patterns and microbial-associated molecular patterns [3]. Damage-associated molecular patterns are endogenous molecules released in the extracellular environment following exposure to stressors and tissue damage, including extracellular adenosine triphosphate (ATP), circulating uric acid, heat shock proteins, and oxidized molecules [25]. Microbial-associated molecular patterns are conserved structural components of microorganisms, including the non-pathogenic commensal bacteria that can leak from the gut into the peripheral circulation, such as lipopolysaccharides (LPS) [26]. The gut epithelium is an effective barrier, preventing LPS from being absorbed. However, when structural alterations occur in the intestinal epithelium, LPS may leak into the bloodstream, leading to a rise in plasma LPS levels, a condition known as metabolic endotoxemia. Once in the bloodstream, LPS trigger TLR-4, which stimulates the production of proinflammatory cytokines, contributing to systemic low-grade inflammation [27].

These inflammatory inducers bind to pattern recognition receptors expressed on the plasma membrane and within the cytosol of immune cells, initiating inflammatory signaling responses. Patients with psychiatric disorders exhibit increased levels of these receptors, such as TLRs in monocytes and peripheral lymphocytes, and NLRP3 in blood cells and the frontal cortex [28,29,30]. The activation of TLRs occurs through the recruitment of protein kinases, triggering the formation of intracellular protein complexes known as NLRP3 inflammasomes. The stimulation of NLRP3 subsequently activates caspase 1, which cleaves the precursor forms of IL-1β and IL-18 into their active forms. This activation triggers the NF-κB pathway, promoting the transcription of other inflammatory mediators such as IL-6 and TNF-α [31].

Proinflammatory cytokines, chemokines, ROS, nitric oxide, and metalloproteinases can increase the permeability of the blood–brain barrier, compromising its ability to protect the brain from potentially harmful substances and immune cells that would normally be restricted from accessing brain tissue. Specifically, proinflammatory cytokines and oxidative stress modulate blood–brain barrier permeability by affecting the tight junctions of cerebrovascular endothelial cells, while metalloproteinases degrade the endothelial basement membrane essential for blood–brain barrier integrity [4]. Once in the brain, inflammatory mediators activate microglial cells, altering the synthesis, transport, and metabolism of neurotransmitters involved in mood regulation, such as serotonin, dopamine, norepinephrine, and glutamate [32]. Type I and II interferons, IL-1β, and TNF-α can reduce the synthesis and availability of monoamines through several mechanisms: (1) decreasing dopamine levels by reducing the concentration of tetrahydrobiopterin, a cofactor for tyrosine hydroxylase, which is essential for dopamine synthesis [33]; (2) decreasing serotonin levels by activating the enzyme indoleamine 2,3-dioxygenase, which breaks down tryptophan, the primary precursor of serotonin, into kynurenine [34]; and (3) increasing monoamine reuptake by enhancing the expression and activity of presynaptic reuptake transporters, stimulated by MAPK activation, leading to reduced availability of these neurotransmitters in synaptic clefts [35].

In addition to these mechanisms, the activation of microglial cells may increase glutamate concentration by stimulating its release from astrocytes, induced by reactive oxygen and nitrogen species, and by converting kynurenine into quinolinic acid, which binds to glutamate receptors, thereby increasing its availability [36]. Excessive glutamate, particularly when associated with extrasynaptic receptors, can lead to a reduction in brain-derived neurotrophic factor, compromising neuronal integrity, neurogenesis, and neuroplasticity [37].

Another mechanism that links the inflammatory markers to mental disorders is related to the activation of the hypothalamic–pituitary–adrenal axis by psychosocial and biological stressors, leading to the increased release of glucocorticoids [38]. These hormones bind to the glucocorticoid receptors, leading to its activation, which in turn translocate to the nucleus and repress proinflammatory signaling pathways, including the NF-κB, and upregulate the expression of anti-inflammatory genes, such as IL-10 [3]. However, chronic exposure to increased cortisol levels results in resistance to the suppressive inflammatory effects of glucocorticoids [5]. Activation of the NLRP3 inflammasome leads to the caspase-mediated cleavage of the glucocorticoid receptor, resulting in a decreased number of this receptor and reduced affinity for their ligands [39]. Stress-induced glucocorticoid resistance represents a biological abnormality observed in patients with major depressive disorder, and it has been associated with systemic low-grade inflammation [40].

In the present study, we found that an elevated concentration of CRP was associated with higher odds of CMD. CRP is an acute-phase protein produced mainly by the liver during acute inflammation [41]. Its gene expression in hepatocytes and subsequent biosynthesis are primarily regulated at the transcriptional level by IL-6 [41]. This acute-phase protein is a widely used marker of inflammation in studies investigating systemic low-grade inflammation due to its ease of clinical application [42]. Elevated CRP concentrations in the central nervous system may promote inflammatory responses, potentially leading to the development of mental disorders. Higher CRP concentrations disrupt the blood–brain barrier by binding to specific ligands, including Fc gamma receptors (CD16 and CD32), which are expressed on microglia, astrocytes, and endothelial cells, thereby increasing blood–brain barrier permeability [43]. As previously mentioned, CRP synthesis is partially induced by IL-6, an interleukin that has been associated with CMD. However, this association was not confirmed in our study. These differing results may be explained by the distinct metabolism of these markers. CRP has a longer half-life, making it a more stable biomarker than IL-6, which fluctuates rapidly in response to transient inflammatory stimuli [5]. Consequently, IL-6 may be more indicative of acute inflammatory processes, which might not correlate as strongly with the chronic nature of psychiatric conditions [5]. 

Findings from the Hispanic Community Health Study indicated an association between anxiety and depressive symptoms and CRP concentrations in diverse Latino populations [44]. Moreover, a systematic review of observational studies found that higher CRP concentrations were linked to greater severity of depressive symptoms, characterized by a specific pattern including fatigue, restless sleep, low energy, concentration difficulties, poor psychomotor speed, poor executive functioning, somatic symptoms, and a worse response to treatment [43]. In the context of anxiety disorders, a meta-analysis demonstrated that CRP concentrations were significantly higher in individuals with generalized anxiety disorder compared to controls [45]. Additionally, a cross-sectional study within the UK Biobank found an association between CRP concentrations greater than 3 mg/L and both panic and generalized anxiety disorders [46].

**Figure 1:**
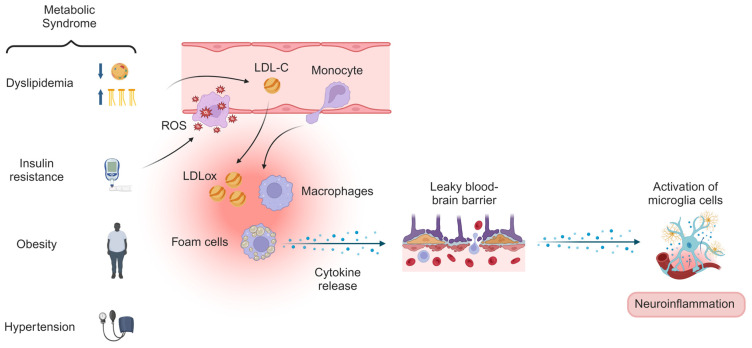
Mechanisms of metabolic syndrome, insulin resistance, and dyslipidemia on neuroinflammation. The combination of insulin resistance and dyslipidemia may lead to vascular damage, which promotes the formation and progression of atherosclerosis and contributes to systemic low-grade inflammation. Peripheral inflammatory mediators can cross the leaky blood–brain barrier, activating microglial cells in the brain. This activation results in neuroinflammation and disrupts the synthesis, transport, and metabolism of neurotransmitters involved in mood regulation, such as serotonin, dopamine, norepinephrine, and glutamate, thereby increasing the risk of mental disorders. Abbreviations: ROS, reactive oxygen species; LDL-C, low-density lipoprotein cholesterol; LDLox, oxidized low-density lipoprotein. Created with BioRender.com.

We observed that participants with insulin resistance, dyslipidemia, and MetS showed higher plasma CRP concentrations than those without these metabolic conditions. Insulin resistance and dyslipidemia are interrelated conditions that significantly contribute to inflammation [6]. Insulin resistance results in elevated blood levels of glucose and lipids, promoting the production of ROS through the activation of enzymes, such as nicotinamide adenine dinucleotide phosphate oxidase and xanthine oxidase [47]. The oxidative stress damages vascular endothelial cells, leading to endothelial dysfunction and increased expression of chemokines and adhesion molecules, including ICAM-1 and VCAM-1. These molecules facilitate the attachment of circulating monocytes to the vascular wall, where they differentiate into macrophages and release proinflammatory cytokines such as IL-1β and TNF-α [6]. Dyslipidemia, characterized by high LDL-c and low HDL-c levels, further exacerbates the inflammatory state. The LDL-c penetrates the subendothelial space where it is modified into oxidized LDL. Oxidized LDL is taken up by macrophages, leading to the formation of foam cells and the activation of the NLRP3 inflammasome, which increases the release of proinflammatory cytokines [48] (Figure 1).

Plasma inflammatory markers may represent a potential clinical biomarker for psychiatric disorders that could be integrated into routine practice to describe the clinical profile and guide clinicians toward personalized treatment [43]. Furthermore, anti-inflammatory interventions based on modifiable lifestyle characteristics, such as dietary patterns and physical activity, may represent a potential strategy in public health interventions to reduce the inflammatory state and consequently prevent the odds of developing CMD. The modulation of the inflammatory state through diet can occur both via the direct action of nutrients and bioactive compounds on inflammatory pathways, as well as through its influence on the promotion or prevention of inflammation risk factors, such as obesity [49]. Previous evidence indicates that some unhealthy dietary patterns, such as the Western diet, are related to the onset and severity of mental disorders [50,51]. In contrast, dietary patterns characterized by high consumption of vegetables, fruits, whole grains, nuts, low-fat dairy, fish, and unprocessed meats—such as the Mediterranean diet—have been associated with protective effects against these conditions [52,53]. Moreover, physical activity has been shown to have a protective effect against systemic low-grade inflammation, since it has been associated with a decrease in proinflammatory cytokines such as IL-6, TNF-α, and CRP while promoting the release of anti-inflammatory mediators like IL-10 and adiponectin [54]. These effects can be explained by exercise-induced improvements in body composition, insulin sensitivity, and immune function [55].

The present findings could be relevant for developing new therapeutic approaches and enhancing our understanding of inflammatory biomarkers in mental disorders, positioning CRP as a possible biomarker for mental disorders. Yet, the present study has some limitations that should be considered. The cross-sectional design limitspotential inference of causality from the associations observed in the findings. Despite the analysis of data derived from a representative sample of the São Paulo population, the results cannot be generalized due to the sampling method for inflammatory and cardiometabolic data collection. Hence, future studies should consider investigating these hypotheses in other populations with alternative study designs.

## 5. Conclusions

Our findings suggest that increased plasma CRP concentrations may be a potential risk factor for CMD. Furthermore, individuals with insulin resistance, dyslipidemia, and MetS demonstrated higher plasma CRP concentrations compared to participants without these diagnoses. Future interventional studies should explore these hypotheses acrossdiverse populations to evaluatethe potential for public health interventions targetingmodifiable lifestyle factors, such asphysical activity and dietary patterns.

## Figures and Tables

**Table 1:** Characterization of the study sample participating in 2015 ISA-Nutrition (n = 575).

Variables (n)	n (%)/Median (Q1–Q3)
**Age (years) (575)**	59 (43–68)
**Sex assigned at birth (575)**	
Male	290 (50.4)
Female	285 (49.6)
**Alcohol (574)**	
No	402 (70)
Yes	172 (30)
**Smoking (575)**	
No	479 (83.3)
Yes	96 (16.7)
**Physical activity (564)**	
Minutes/week	480 (160–1205)
**Nutritional status (570)**	
Underweight	61 (10.7)
Normal weight	212 (37.2)
Overweight	144 (25.3)
Obese	153 (26.8)
**Type 2 diabetes (575)**	
No	453 (78.8)
Yes	122 (21.2)
**Systemic arterial hypertension (570)**	
No	259 (45.4)
Yes	311 (54.6)
**Dyslipidemia (566)**	
No	164 (29)
Yes	402 (71)
**Common mental disorders (575)**	
No	445 (77.4)
Yes	130 (22.6)

Data are presented in absolute and relative frequencies or median and interquartile range (Q1–Q3).

**Table 2:** Comparison of plasma inflammatory biomarkers concentrations according to the presence of common mental disorders in adults and older adults of the 2015 ISA-Nutrition (n = 575).

Inflammatory Biomarkers	Common Mental Disorders		*p*-Value
	No	Yes	
IL-6 (pg/mL)	1.40	1.30	0.71
IL-1β (pg/mL)	1.13	1.15	0.38
IL-10 (pg/mL)	4.02	4.15	0.24
TNF-α (pg/mL)	11.10	11.16	0.77
PAI-1 (ng/mL)	23.66	29.11	0.02 *
MCP-1 (ng/mL)	0.29	0.31	0.36
sICAM-1 (ng/mL)	245	258	0.09
sVCAM-1 (ng/mL)	806	813	0.24
CRP (mg/L)	0.35	0.46	0.01 *
Leptin (ng/mL)	1.76	2.31	0.08
Adiponectin (ng/mL)	19,212	18,340	0.80
SIS	1.29	1.47	0.02 *

Data are presented in the median. * Significant difference in the Mann–Whitney test. Abbreviations: IL, interleukin; TNF-α, tumor necrosis factor-alpha; MCP-1, monocyte chemotactic protein-1, PAI-1, plasminogen activator inhibitor-1; sICAM-1, soluble intercellular adhesion molecule-1; sVCAM-1, soluble vascular cell adhesion molecule-1; CRP, C-reactive protein; SIS, systemic low-grade inflammation score.

**Table 3:** Association between plasma C-reactive protein and plasminogen activator inhibitor 1 levels, the systemic low-grade inflammation score, and common mental disorders in adults and older adults in 2015 ISA-Nutrition (n = 575).

Inflammatory Biomarkers	OR	95% CI	*p*-Value
CRP (mg/L)	1.54	1.09–2.19	0.01 *
PAI-1 (ng/mL)	1.00	1.00–1.02	0.14
SIS	1.43	0.96–2.11	0.08

Data are presented in odds ratio (OR) and 95% confidence interval (95% IC). * Significant difference in the multivariable logistic regression model adjusted for age, sex assigned at birth, body mass index, weekly physical activity, alcohol consumption, smoking, type 2 diabetes, systemic arterial hypertension, and dyslipidemia diagnosis. Abbreviations: CRP, C-reactive protein; PAI-1, plasminogen activator inhibitor-1; SIS, systemic low-grade inflammation score.

**Table 4:** Comparison of plasma C-reactive protein concentrations according to cardiometabolic markers in adults and older adults of the 2015 ISA-Nutrition (n = 575).

Cardiometabolic Markers (n)	CRP (mg/L)	*p*-Value
**Fasting glycemia**		0.38
<100 mg/dL (385)	0.37	
100–126 mg/dL (190)	0.40	
**HOMA-IR**		<0.001 *
<2.71 (289)	0.30	
>2.71 (285)	0.53	
**Serum triglycerides**		<0.001 *
<150 mg/dL (404)	0.32	
>150 mg/dL (160)	0.56	
**Total cholesterol**		0.05
<190 mg/dL (334)	0.34	
>190 mg/dL (230)	0.43	
**LDL-c**		0.25
<130 mg/dL (396)	0.37	
>130 mg/dL (167)	0.41	
**HDL-c**		<0.001 *
Male > 40 mg/dL (154) Female > 50 mg/dL (109)	0.31	
Male < 40 mg/dL (130) Female < 50 mg/dL (170)	0.43	
**non-HDL-c**		0.03 *
<160 mg/dL (405)	0.35	
>160 mg/dL (158)	0.45	
**Metabolic syndrome**		<0.001 *
Yes (186)	0.42	
No (185)	0.27	

Data are presented in the median. * Significant difference in the Mann–Whitney test. Abbreviations: HOMA-IR, Homeostasis Model Assessment for Insulin Resistance; LDL-c, low-density lipoprotein cholesterol; HDL-c, high-density lipoprotein cholesterol; non-HDL-c, non-high-density lipoprotein cholesterol.

## Data Availability

The dataset presented in this article is not readily available because the data are part of an ongoing study. Requests to access the dataset should be directed to R.M.F.
